# Veterinary Surgery

**Published:** 1844-04

**Authors:** 


*Veterinary Surgery.*—We now pass on to notice a very remarkable form of hepatic disease in the horse, which well deserves the notice of the human pathologist.

It appears that, although a horse seems to be in perfect health, and is moreover capable of laborious work, the liver may have become so softened and pulpy and its peritoneal covering so loosely adherent to its parenchyma, that a sudden exertion will cause a rupture of the viscus, and an immense extravasation of blood into the abdomen, to take place. The accident, as a matter of course, is generally fatal, and the death is sudden. The curious circumstance in this disease is that, in most instances, there are no premonitory symptoms of any hepatic derangement; the animal often has been remarkably healthy, the biliary secretion regular, and the alvine excretions natural. When the extravasation is large and rapid, the symptoms are those of internal hæmorrhage—pulse irregular and indistinct; action of heart tumultuous; profuse sweating; pawing; curling of the upper lip and pouting of the nose; lifting the hind legs to the belly; hurried respiration and deep and frequent sighing; distended abdomen; blanched conjunctiva and buccal membrane; amourosis; extreme debility; fainting and death.

In some cases, however, although the parenchyma of the liver has degenerated into one huge mass of bloody pulp, its peritoneal coat remains entire, or is only slightly ruptured. The symptoms then are much more indistinct than those we have just enumerated, and often far from being satisfactory, as regards the diagnosis. Weakness and faintness at work, diminished appetite, swelling of the hind legs, fulness of the abdomen, buccal and Schneiderian membranes pale, pulse frequent and feeble, occasional sighing, partial amaurosis, with dilated pupil of one or both eyes—these are the most common features of the case.

*Purpura Hæmorrhagica* in the horse is characterised by the occurrence of petechial and ecchymosed spots and patches in almost every part and tissue of the body—on the skin, the Schneiderian membrane, the conjunctivæ of the eyes, under the fasciæ and in the substance of the muscles, and occasionally also in the lungs, kidneys, and other viscera. The animal is uneasy, and in pain; his breathing is usually hurried and snuffling; and his pulse is rapid and wiry, or full and strong. As the disease advances, the echymosed swellings become confluent; a sero-sanguineous discharge drips from the nostrils; the Schneiderian membrane is sometimes nearly black; the circulation is more frequent and feeble; *the horse paws, or looks back, or anxiously turns his head from side to side* (a graphic description;) he totters, if made to walk about; becomes weaker, and dies. If blood be drawn, it is found to be thin and pallid, and separates rapidly into its two portions. The urine occasionally contains much albumen, and coagulates by nitric acid and heat: sometimes it is mixed with blood.

As Mr. Field remarks, the disease is seemingly referrible to a morbid condition—characterised chiefly by an increased tenuity—of the circulating fluids. There may indeed be present, at the same time, an unusual laxity and dilatability of the blood-vessels, with a certain degree of obstruction in the capillaries, and an augmented impetus in the heart's action; but certainly the primary and essential feature of Purpura is an alteration in the composition and vital properties of the blood itself. This opinion is confirmed by the circumstance of its usual exciting causes being general debility, poor living, unwholesome diet, and excessive labor.

As to the *treatment*, bleeding may be occasionally necessary, when vital organs are affected or threatened; "but it rarely (does it ever?) benefits." Turpentine is useful; so are the sulphate of iron, the mineral acids, &c. The cold effusion is "particularly good." The pure air of the country, with grass feeding, is the best restorative.

In a fatal case of Hæmaturia, the peritoneal coat and cortical substance of the kidney (kidneys?) were entirely destroyed. The ureters were filled with blood: the bladder contained about two pints, mixed with urine. The liver was very pale; the intestines healthy. About a pint of blood was found within the pericardium. In another fatal case, where the quantity of blood lost had been enormous, the tubuli uriniferi and pelvis of the left kidney were found ulcerated, and the texture of the right one was soft and flabby.

*Lithotomy.*—The symptoms of calculus in the bladder are, as might be supposed, very much alike to those that are observed in the human subject. We read of the animal "staling blood after work,—continually making attempts to stale—straining violently and voiding only a small quantity at a time—and the urine being turbid and alkalescent." The actual presence of the stone is ascertained by an examination *per anum*, and by the introduction of a catheter or sound into the bladder. The following account of the mode, in which a horse is cut for the stone, is well worth a perusal: we cannot abridge it without injury.

"The animal was cast in the usual manner, and both hind legs were drawn to the shoulders, as if for castration. Read's new flexible catheter being passed into the bladder, a quantity of warm water was injected, sufficient to distend that organ and the urethra moderately. The catheter being withdrawn, and holding the penis with the left hand, a slightly-curved groove staff, two feet long, was introduced, so as for the curved part to come into the sub-anal portion of the urethra, above the posterior edge of the ischium, extending towards the sphicter ani. An assistant kneeling on the left side of the horse, drew the penis forwards with his left hand, and gently pushed the staff backwards with the right, at the same time keeping the groove exactly beneath the *raphe*; this elevated the portion of the urethra to be incised. I then made an incision, a line from and on the right side of the *raphe*, through the skin and fascia, extending the length of it from three to four inches; and, pushing the penis a little on one side, I gradually divided the muscular and spongy portion, and exposed the mucous membrane of the urethra, when the finger readily detected the groove of the staff, into which a small incision was made sufficient to admit the *bistoire cachée*, following which with the index finger of the left hand, the membrane was divided to the rectum. Very little blood flowed, and the water of the urethra gushed out. The staff being removed, I easily introduced the small forceps through the urethra into the bladder, and grasped the stone, a portion of which flaked off. The large forceps were then employed, and, my brother holding the handles, I directed the blades upon the stone, my left hand being in the rectum. Having placed the stone in a proper position, I grasped it with the forceps, and with both hands gave it a half-turn, so as to place its widest axis between the pubis and rectum; and thus, with a moderate force, I gradually and evenly drew it out, the neck of the bladder readily dilating. Two stitches were inserted in that part of the incision nearest the anus, the lower part being left to itself."

The calculus consisted entirely of carbonate of lime and animal matter. The animal did well.—*Med. Chi. Rev.*, Jan.

